# Effects of Fermented Milk Containing *Bifidobacterium animalis* Subsp. *lactis* MN-Gup (MN-Gup) and MN-Gup-Based Synbiotics on Obesity Induced by High Fat Diet in Rats

**DOI:** 10.3390/nu14132631

**Published:** 2022-06-24

**Authors:** Chenyuan Wang, Shusen Li, Erna Sun, Ran Xiao, Ran Wang, Yimei Ren, Jingjing He, Qi Zhang, Jing Zhan

**Affiliations:** 1 Key Laboratory of Precision Nutrition and Food Quality, Department of Nutrition and Health, China Agricultural University, Beijing 100193, China; drwangchenyuan@163.com (C.W.); xiaoran881230@163.com (R.X.); renyimei123@cau.edu.cn (Y.R.); hejingjing_89@163.com (J.H.); zhangqi@cau.edu.cn (Q.Z.); 2 Mengniu Hi-tech Dairy Product Beijing Co., Ltd., Beijing 101100, China; lishusen@mengniu.cn (S.L.); sunerna@mengniu.cn (E.S.); 3 Key Laboratory of Functional Dairy, Department of Nutrition and Health, China Agricultural University, Beijing 100193, China; wangran@cau.edu.cn

**Keywords:** *Bifidobacterium animalis* subsp. *lactis* MN-Gup, obesity, gut microbiota, galacto-oligosaccharides, xylo-oligosaccharides

## Abstract

Given the probiotic effects previously found in *Bifidobacterium animalis* subsp. *lactis* MN-Gup (MN-Gup) and its great application potential in dairy products, this study aimed to investigate the effects of fermented milk containing MN-Gup or MN-Gup-based synbiotics on high fat diet (HFD)-induced obesity in rats. Galacto-oligosaccharides (GOS) and xylo-oligosaccharides (XOS) were selected as the tested prebiotics in MN-Gup-based synbiotics due to their promotion of MN-Gup growth in vitro. After nine weeks of HFD feeding, the obese rats were intervened with fermented milk containing MN-Gup (MN-Gup FM) or its synbiotics (MN-Gup + GOS FM, MN-Gup + XOS FM) for eight weeks. The results showed that the interventions could alleviate HFD-induced body weight gain, epididymal fat deposition, adipocyte hypertrophy, dyslipidemia and inflammation, but GOS and XOS did not exhibit significant synergies with MN-Gup on those alleviations. Furthermore, the interventions could regulate the HFD-affected gut microbiota and microbial metabolites, as shown by the increases in short chain fatty acids (SCFAs) and alterations in obesity-related bile acids (BAs), which may play important roles in the mechanism underlying the alleviation of obesity. This study revealed the probiotic effects of MN-Gup on alleviating obesity and provided the basis for MN-Gup applications in the future.

## 1. Introduction

The prevalence of overweight and obesity has become a serious health problem in the world. Obesity is usually associated with a high risk of chronic diseases, such as type 2 diabetes, gallbladder disease, cardiovascular disease and cancer [1,2]. People continue to seek various methods to alleviate obesity, such as diet regulation, physical exercise and bariatric surgery [3]. Recently, obesity has been found to be correlated with an imbalance in gut microbiota, and gut microbiota has contributed to a new perspective for regulating host energy metabolism [4]. A number of mechanisms have been proposed for gut microbiota-influenced obesity [5]. For instance, some microbiota can promote the production of short chain fatty acids (SCFAs), which can activate G protein-coupled receptors and induce subsequent intestinal hormone secretion to regulate energy metabolism [6].

Probiotics, defined as “live microorganisms which give a health benefit to the host when administered in adequate amounts” [7], have exerted excellent effects on modulating gut homeostasis via balancing beneficial and harmful bacteria, improving intestinal barrier function, and regulating immune responses [8]. It is estimated that the probiotics market grew 37% globally from 2016 to 2020 [9]. Accumulating animal and human data have shown that probiotics exhibit great potential in anti-obesity applications [8]. *Bifidobacterium lactis* and *Lactobacillus paracasei* are common probiotics that have been reported to regulate gut microbiota and attenuate obesity in high fat diet (HFD)-fed mice [10,11,12]. Meanwhile, the effects of probiotics or their applications in food on obesity were investigated in randomized controlled trials (RCTs). For example, daily consumption of fermented milk containing 2 × 10^9^ CFU of *Lactobacillus gasseri* SBT2055 for 12 weeks was found to significantly reduce body mass index (BMI), waist, and hip circumferences in Japanese adults with large visceral fat areas [13]. A meta-analysis study based on twenty-six RCTs showed that there were significant effects of probiotics on reducing body weight, waist circumference, fat mass, total cholesterol (TC), tumor necrosis factor-α (TNF-α) level of overweight/obese subjects [14]. Therefore, the dietary intervention using probiotics may provide a potential strategy to alleviate obesity.

Furthermore, prebiotics, some non-digestible food ingredients that can selectively stimulate the growth and/or activity of beneficial bacteria in the intestinal tract, have been suggested to regulate gut microbiota and exhibit a synergy with probiotics [15]. As a result, the combinations of probiotics and prebiotics, namely synbiotics, have attracted extensive attention as tools to help humans maintain optimal health [16]. To date, prebiotics such as inulin, fructo-oligosaccharides (FOS), galacto-oligosaccharides (GOS), xylo-oligosaccharides (XOS) and polydextrose have been commonly used with probiotics as synbiotics in obesity treatment [17,18]. For example, a synbiotic containing *Lactobacillus fermentum* CECT5716 and FOS could reverse HFD-induced gut microbial dysbiosis and metabolic syndrome in rats [19]. A clinical study showed that *Bifidobacterium animalis* subsp. *lactis* 420 with polydextrose could alter gut microbiota and microbial metabolism, which may support the alleviation of obesity [20]. Synbiotic supplementation could not only reduce body weight, but also improve obesity-related phenotypes like blood lipids, cytokines, and oxidative stress [21]. Thus, it is of great significance to explore synbiotics for alleviating obesity.

*Bifidobacterium animalis* subsp. *lactis* MN-Gup (MN-Gup), an aerospace mutant and screened according to high oxygen tolerance coefficient from the Shenzhou-11 re-entry spacecraft, has been demonstrated to benefit health associated with gut microbiota [22]. Given the wide applications of probiotics in fermented milk, the current study aimed to investigate the effects of fermented milk containing MN-Gup or MN-Gup-based synbiotics on alleviation of obesity in HFD-fed rats and elucidate the underlying mechanism from the perspectives of gut microbiota and its metabolites. This study will help to explain the anti-obesity function of MN-Gup and its synbiotics, and provide the theoretical bases for the applications of MN-Gup in foods.

## 2. Materials and Methods

### 2.1. Bacterial Strain and Its Prebiotics Screening In Vitro

*Bifidobacterium animalis* subsp. *lactis* MN-Gup (MN-Gup, CGMCC No. 15578) was provided by Mengniu Hi-tech Dairy Product Beijing Co., Ltd. (Beijing, China). The strain was activated in De Man, Rogosa, Sharpe (MRS) broth (Difco Laboratories, Detroit, MI, USA) and then incubated at 37 °C for 24 h and repeated three times. Individual prebiotics including fructo-oligosaccharides (FOS), resistance dextrin (RD), inulin, xylo-oligosaccharides (XOS), and galacto-oligosaccharides (GOS) were purchased from Baoling Bao Biology Company (Dezhou, China) and sterilized before use. The prebiotic index (PI) was used to evaluate the relative growth-promoting capability of a prebiotic to that of glucose [23]. The activated MN-Gup was placed in the MRS containing glucose or 1.5% prebiotics as a carbon source, or in a glucose-free MRS medium, and statically cultivated at 37 °C. The absorbance of the fermentation broth was measured at 660 nm at 0 h, 4 h, 8 h, 12 h, and 24 h, respectively, and the PI was calculated according to the following formula.

$$\mathrm{PI}=\frac{\left(A_{\mathrm{PP}24}-{\;A}_{\mathrm{PP}0}\right)-{\;(A}_{\mathrm{PN}24\;}-{\;A}_{\mathrm{PN}0})}{\left(A_{\mathrm{PG}24}-{\;A}_{\mathrm{PG}0}\right)-{\;(A}_{\mathrm{PN}24}-{\;A}_{\mathrm{PN}0})}$$

A_PP24_ and A_PP0_ represent the absorbance of fermentation broth of MN-Gup cultured for 24 h and 0 h when the carbon source is a prebiotic in medium; A_PG24_ and A_PG0_ represent the absorbance of fermentation broth of MN-Gup cultured for 24 h and 0 h when the carbon source is glucose in medium; A_PN24_ and A_PN0_ represent the absorbance of fermentation broth of probiotics strains cultured for 24 h and 0 h in a glucose-free MRS medium.

### 2.2. Preparation of the Test Fermented Milk

Fermented milk was prepared using a commonly used procedure for conventional production of fermented milk with a modification [13], and the main ingredients of fermented milk were provided by Mengniu Hi-tech Dairy Product Beijing Co., Ltd. (Beijing, China). The test fermented milk was added with 1 × 10^8^ CFU/g of MN-Gup or synbiotics consisting of 1 × 10^8^ CFU/g of MN-Gup with either 1.5% GOS or 1.5% XOS. Erythritol was used as calorie-free sweetener in the fermented milk, and per 100 g of fermented milk contained 3.0 g protein, 3.5 g fat, and 5.3 g carbohydrate and its total energy was 78 kcal. The test fermented milk was kept in cold storage and delivered weekly.

### 2.3. Animals and Experimental Design

Male SD rats (six weeks old) were purchased from Charles River (Beijing, China) and housed in an SPF-grade laboratory animal facility under a standard light/dark (12 h/12 h) cycle with free access to water and food in Pony Testing International Group Co., LTD (Beijing, China). The study was approved by the institutional animal ethics committee (Approval Number: PONY-2021-FL-16). After a one-week acclimatization period, all rats were divided into five groups, and each group consisted of five rats. One group was continuously fed with a normal-chow diet (ND group) and the other four groups of rats were changed to a refined HFD diet (45% fat, TROPHIC, Nantong, China). After nine weeks, HFD induced obesity in rats based on 15% greater body weight gain than ND group, and three groups of obese rats were intragastrically administered with 2 mL the test fermented milk containing MN-Gup (MN-Gup FM), MN-Gup FM with GOS (MN-Gup + GOS FM), and MN-Gup FM with XOS (MN-Gup + XOS FM), and the other group of HFD-induced obese rats was intragastrically administered 2 mL of 0.9% sterile saline (HFD group). The body weight of rats was recorded once a week, and the food intake was replaced and recorded twice a week. After eight weeks of intervention, the rats were euthanised and their blood samples were collected to prepare serum. The epididymal fat pads were weighed and fixed in 4% paraformaldehyde, and colonic feces were also collected and stored at −80 °C until analysis.

### 2.4. Biochemical Parameters Analysis in Serum

The levels of triglycerides (TG), total cholesterol (TC), low-density lipoprotein (LDL) and high-density lipoprotein (HDL) cholesterol in serum were determined by commercial kits according to the manufacturer’s recommendations. Cytokines concentration of tumor necrosis factor-α (TNF-α), interleukin (IL)-6, IL-4 and IL-10 were measured by commercial enzyme-linked immunosorbent assay (ELISA) kits.

### 2.5. Histological Analysis

The fixed epididymal fat tissues were embedded in paraffin and stained with haematoxylin and eosin (H&E). Finally, the histopathology of epididymal adipocyte was visualized using an optical microscope (DMi8, Leica, Weztlar, Germany), and adipocyte size was analyzed by Image-Pro Plus 6.0 software (Media Cybernetics, Silver Spring, MD, USA) according a previous study.

### 2.6. Gut Microbiota Analysis

Feces bacterial analysis was performed on the V3-V4 region of the 16S rRNA gene using Illumina MiSeq sequencing as described previously [22]. Briefly, DNA was extracted from the fecal samples using soil DNA extraction kits (Omega Bio-tek, Norcross, GA, USA) according to the manufacturer’s protocols. The hypervariable region V3-V4 of 16S rRNA genes were amplified by PCR using the primers 338F (5′-GTACTCCTACGGGAGGCAGCA-3′) and 806R (5′-GTGGACTACHVGGGTWTCTAAT-3′). Sequencing of the PCR amplification products was performed on an Illumina Miseq platform (PE300). Operational taxonomic units (OTUs) were clustered using UPARSE (version 7.1) based on 97% similarity. The taxonomy of each 16S rRNA gene sequence was analyzed by an RDP Classifier algorithm.

### 2.7. Measurement of Short Chain Fatty Acids (SCFAs) in Colonic Feces

Acetate, propionate and butyrate were measured as previously described [24]. Briefly, 20 mg of fecal sample was acidified with 50 μL of 50% sulfuric acid and homogenised with 500 μL of diethyl ether (containing 19.8 mM 2-ethylbutyric acid as the internal standard) for 1 min using a Mini Bead beater-16 (BioSpec Products, Inc., Bartlesville, OK, USA). Then, the samples were centrifugated at 14,000 rpm for 5 min, and then the supernatant was collected. Extraction was repeated, and the supernatant was combined. After filtering through a 0.22 μm membrane, SCFA content was detected by a gas chromatograph (GC, Shimadzu, GC-2010 Plus, Kyoto, Japan) equipped with the DB-FFAP column (30 m × 0.25 mm × 0.25 μm, Agilent Technologies, Inc., Santa Clara, CA, USA) and FID detector. The splitless injector temperature was 250 °C and the injection volume was 1 μL. The carrier gas was nitrogen. The GC oven temperature program was set as below: maintained at 60 °C for 4 min, increased by 20 °C/min to 180 °C for 1 min, then increased by 50 °C/min to 220 °C for 1 min. The temperature of the FID detector was maintained at 250 °C.

### 2.8. Measurement of Bile Acids (BAs) in Colonic Feces

An aliquot of 15 mg fecal sample was homogenised with 1 mL of ethanol containing 5% ammonia water and isotope internal standards for five cycles of 15 s. After centrifugation at 14,000 rpm for 10 min at 4 °C, the supernatant was collected. Extraction was repeated three times, and the supernatant was combined and dried under a gentle nitrogen flow. The residue was accurately re-dissolved with 1.8 mL of solution consisting of acetonitrile and water (75:25, *v:v*), and finally passed through a 0.22 μm of filter for UPLC-Q-TOF (G6540, Agilent Technologies, Inc., Santa Clara, CA, USA) analysis. Bile acids were separated with a BEH C_18_ (2.1 mm × 100 mm, 1.7 μm) UPLC column (Waters Inc., Milford, CT, USA), which was kept at 30 °C with a 0.3 mL/min flow rate. The injection volume was 5 μL and the separation was carried out using a gradient elution with water (containing 0.01% formic acid and 2 mM acetic acid) and acetonitrile. The parameters of mass spectrum were set at as follow: electrospray ionization (ESI) model, the detector operated in a low mass range (1700 *m*/*z*) and a 2 GHz extended dynamic range, the MS scan range was 100–1200 *m*/*z*, and the acquisition rate was two spectrum per second. Twenty-four BAs presented in Table S1 were identified and quantified, but the concentrations of some BAs were too low to reach the detection limits. The detectable BAs included taurocholic acid (TCA), alpha-muricholic acid (α-MCA), tauro-alpha-muricholic acid (T-α-MCA), beta-muricholic acid (β-MCA), tauro-beta-muricholic acid (T-β-MCA), omega-muricholic acid (ω-MCA), deoxycholic acid (DCA), taurodeoxycholic acid (TDCA), hyodeoxycholic acid (HDCA) and tauroursodeoxycholic acid (TUDCA).

### 2.9. Statistical Analysis

Data were shown as means ± standard deviations (SD). Statistical analysis was carried out using SPSS software (version 23.0, Chicago, IL, USA). One-way ANOVA with Duncan’s post hoc test was used to evaluate differences among groups, and significant difference was set at *p* < 0.05.

## 3. Results

### 3.1. Selection of Prebiotics with the Capability to Promote the Growth of MN-Gup In Vitro

FOS, RD, inulin, XOS and GOS are five well-defined prebiotics, and their capability to promote the growth of MN-Gup was investigated. As shown in Figure S1A, the growth of MN-Gup was very slow in the glucose-free medium. While in the medium containing glucose, MN-Gup exponentially grew over time. FOS, RD, inulin, XOS and GOS all promoted MN-Gup growth to varying degrees, indicating they could be used as carbon sources for MN-Gup growth. During the 24-h incubation period, the trend of promotion on MN-Gup growth of XOS was the most similar to that of glucose; and GOS had a weak effect on the growth of MN-Gup before 12 h, but its growth-promoting effect was suddenly accelerated after 12 h (Figure S1A). The prebiotic index (PI) of these five prebiotics was GOS > XOS > inulin > RD > FOS (Figure S1B). Therefore, GOS and XOS were the potential prebiotics that could be applied with MN-Gup in symbiotics, and selected for the following study.

### 3.2. Effects of Fermented Milk Containing MN-Gup or MN-Gup-Based Synbiotics on Body Weight and Body Fat in HFD-Induced Obese Rats

**Figure 1:**
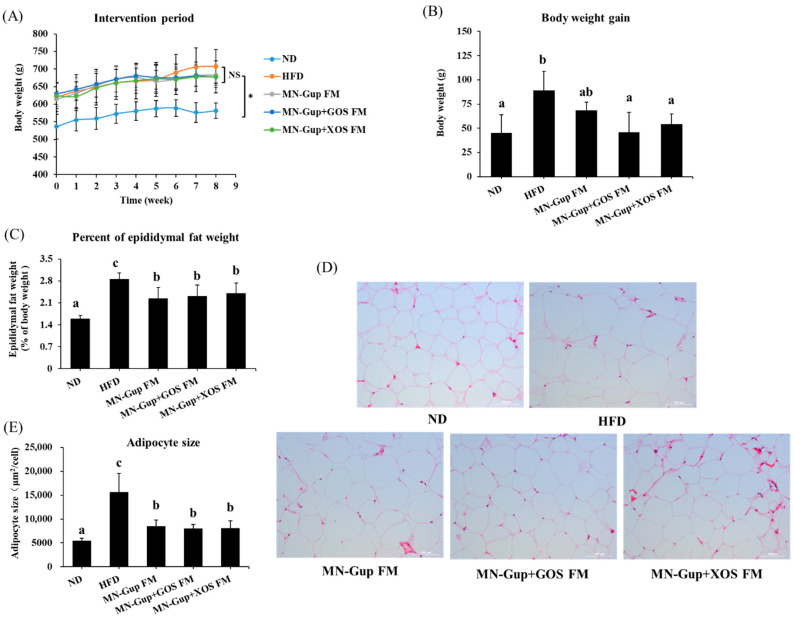
Effects of fermented milk containing MN-Gup or MN-Gup-based synbiotics on body weight and body fat in HFD-fed rats. (**A**) changes of body weight during intervention period (* indicates that ND group is significantly different from other groups); (**B**) body weight gain, (**C**) percent of epididymal fat weight; (**D**) representative images of hematoxylin and eosin (H&E) staining in epididymal adipose tissues; and (**E**) epididymal adipocyte size. Different lowercase letters (e.g., a, b, c) indicate significant differences, *p* < 0.05, (*n* = 5).

As shown in Figure S2A, body weight of all groups of rats had no significant difference before HFD feeding, and the obese rat model was successfully established through a 9-week HFD feeding base on a gain of at least 15% of body weight relative to ND-fed rats. During the intervention period, HFD induced durative body weight gain, and interventions with MN-Gup FM, MN-Gup + GOS FM and MN-Gup + XOS FM curbed the weight gain of rats (Figure 1A), which may be related to the fewer energy intake in intervention groups (Figure S2B). After eight weeks of intervention, HFD-fed rats showed a significant body weight gain compared to ND-fed rats (*p* < 0.05), and MN-Gup FM, MN-Gup + GOS FM and MN-Gup + XOS FM resulted in less body weight gain in HFD-fed rats (Figure 1B). The body weight gains in MN-Gup + GOS FM and MN-Gup + XOS FM group were significantly decreased than that in HFD group (*p* < 0.05), whereas the body weight gain in MN-Gup FM group was not significantly different from that in HFD group (Figure 1B). These results showed that fermented milk containing MN-Gup and MN-Gup-based synbiotics could reverse HFD-induced weight gain, and MN-Gup with GOS and XOS have a better performance that MN-Gup alone on suppressing body weight gain.

Obesity is highly connected to body fat accumulation and adipocyte hypertrophy [25]. HFD significantly increased the percentage of epididymal fat mass, which was reversed by interventions with MN-Gup FM, MN-Gup + GOS FM and MN-Gup + XOS FM (*p* < 0.05, Figure 1C). As shown in Figure 1D,E, HFD caused epididymal adipocyte hypertrophy, and MN-Gup FM, MN-Gup + GOS FM and MN-Gup + XOS FM all significantly reversed the increased adipocyte sizes (*p* < 0.05). These results suggested that fermented milk containing MN-Gup and its synbiotics with GOS or XOS could reduce the body fat induced by HFD, but GOS and XOS did not exhibit a significant synergy with MN-Gup.

### 3.3. Effects of Fermented Milk Containing MN-Gup or MN-Gup-Based Synbiotics on Lipid Profile Levels in HFD-Induced Obese Rats

**Figure 2:**
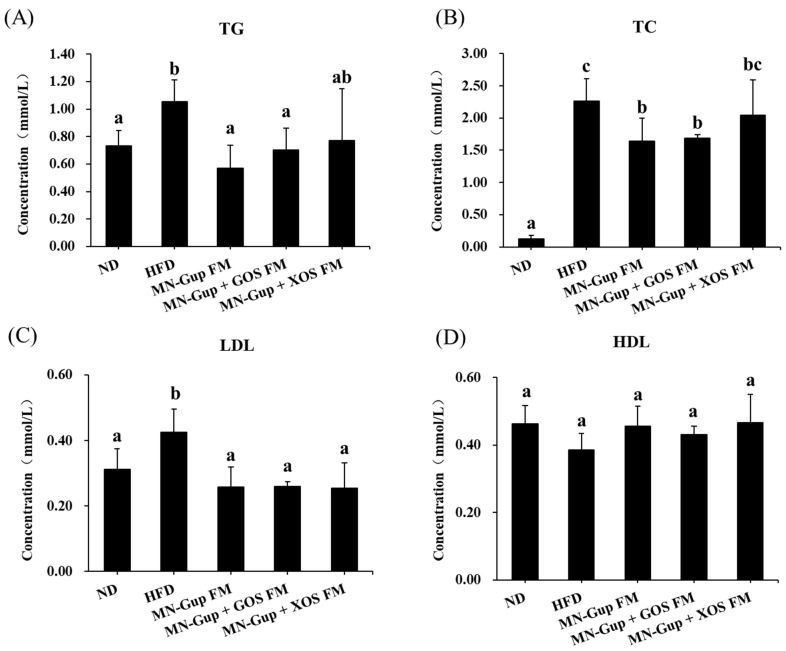
Effects of fermented milk containing MN-Gup or MN-Gup-based synbiotics on lipid profile levels in HFD-induced obese rats. The concentrations of (**A**) triglyceride (TG), (**B**) total cholesterol (TC), (**C**) low-density lipoprotein (LDL) cholesterol, and (**D**) high-density lipoprotein (HDL) cholesterol were measured in rat serum. Different lowercase letters (e.g., a, b, c) indicate significant differences, *p* < 0.05, (*n* = 5).

Dyslipidemia is a common complication of obesity. The levels of TG, TC and LDL were significantly elevated in serum of HFD-fed rats, whereas they were significantly decreased by MN-Gup FM and MN-Gup + GOS FM interventions (*p* < 0.05, Figure 2A–C). MN-Gup + XOS FM did not obviously influence the concentrations of TG and TC compared to HFD group, but resulted in significant down-regulation of LDL concentration (*p* < 0.05). No significant changes were found in HDL levels in all groups (Figure 2D). These results indicated that fermented milk containing MN-Gup and its synbiotics with GOS exerted better effects than its synbiotics with XOS in alleviating dyslipidemia.

### 3.4. Effects of Fermented Milk Containing MN-Gup or MN-Gup-Based Synbiotics on Pro-Inflammatory and Anti-Inflammatory Cytokines in HFD-Induced Obese Rats

**Figure 3:**
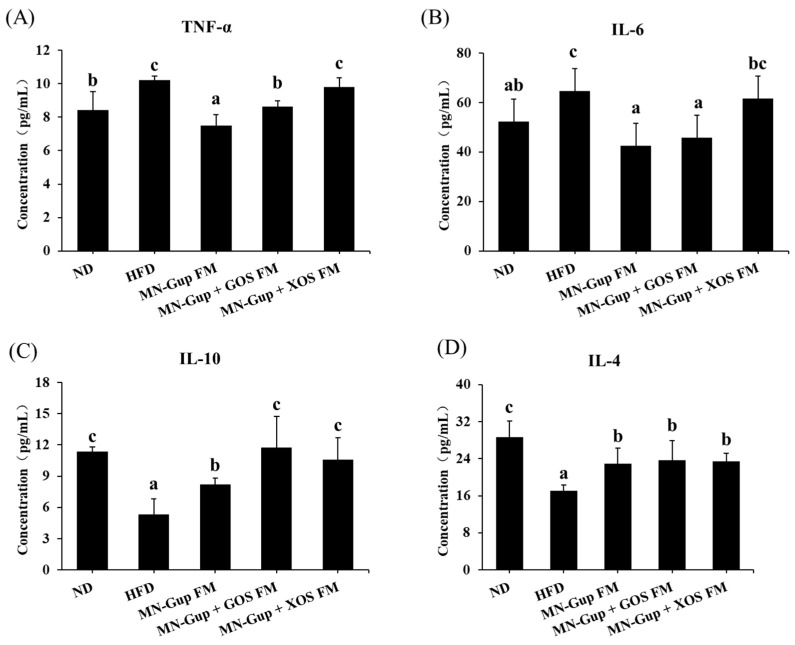
Effects of fermented milk containing MN-Gup or MN-Gup-based synbiotics on pro-inflammatory and anti-inflammatory cytokines in HFD-induced obese rats. The levels of (**A**) tumor necrosis factor α (TNF-α), (**B**) interleukin 6 (IL-6), (**C**) IL-10, and (**D**) IL-4 in serum were measured. Different lowercase letters (e.g., a, b, c) indicate significant differences, *p <* 0.05, (*n* = 5).

Obesity is associated with a chronic low-grade inflammation state of the host [26]. As shown in Figure 3A,B, HFD induced a significant increase in the level of pro-inflammatory cytokines IL-6 and TNF-α and a significant decrease in the level of anti-inflammatory cytokines IL-10 and IL-4 in rat serum, which were significantly reversed by interventions with MN-Gup FM and MN-Gup + GOS FM (*p* < 0.05). MN-Gup + XOS FM had no significant effects on IL-6 and TNF-α levels, but significantly increased IL-4 and IL-10 levels compared to the HFD group (*p* < 0.05). These results indicated that MN-Gup FM and MN-Gup + GOS FM could reverse HFD-induced inflammation by down regulating pro-inflammatory cytokines and up regulating anti-inflammatory cytokines, and MN-Gup + XOS FM may alleviate HFD-induced inflammation by elevating anti-inflammatory cytokines.

### 3.5. Effects of Fermented Milk Containing MN-Gup or MN-Gup-Based Synbiotics on Gut Microbiota in HFD-Induced Obese Rats

**Figure 4:**
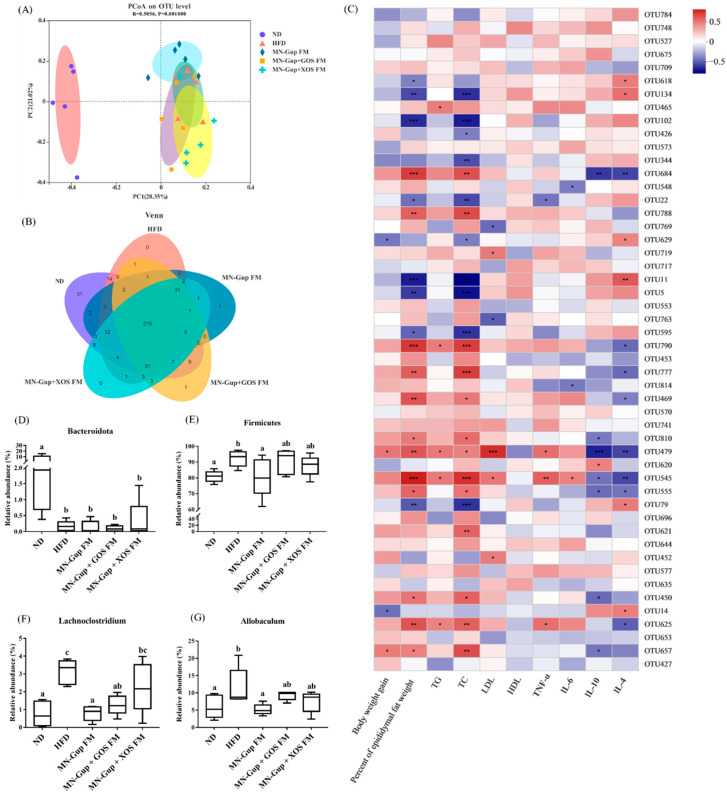
Effects of fermented milk containing MN-Gup or MN-Gup-based synbiotics on gut microbiota in HFD-induced obese rats. (**A**) Principal coordinate analysis (PCoA) based on bacterial operational taxonomic units (OTUs) using Bray-Curtis calculation; (**B**) Venn diagram; (**C**) the heatmap of correlation analysis between gut microbiota (based on OTUs) and obesity related indexes (significant correlations were noted by * *p <* 0.05, ** *p <* 0.01, and *** *p <* 0.001); (**D**,**E**) the significantly altered bacteria at phylum level; (**F**,**G**) the significantly altered bacteria at genus level (different lowercase letters (e.g., a, b, c) indicate significant differences, *p <* 0.05, *n* = 5).

There has been a lot of evidence for the important role of gut microbiota in obesity. The changes in gut microbiota were investigated via 16S rDNA sequencing of colon fecal genes. As shown in Figure 4A, principal coordinates analysis (PCoA) based on operational taxonomic units (OTUs) presented that HFD feeding caused a distinct clustering from ND feeding, indicating that the gut microbiota composition was apparently changed by HFD feeding. The clustering of MN-Gup FM, MN-Gup + GOS FM and MN-Gup + XOS FM groups was different from that of HFD group but has not been completely separated (Figure 4A). A Venn diagram showed that there were 215 OTUs shared in all groups, and 31 OTUs were specific in the ND group and 1 OTU was respectively specific in MN-Gup FM and the MN-Gup + GOS FM group (Figure 4B). The correlations between gut microbiota (based on OTUs) and obesity related indexes (including body weight gain, percent of epididymal fat weight, the levels of TG, TC, HDL, LDL, TNF-α, IL-6, IL-10, and IL-4) were investigated using the Pearson correlation analysis. The heatmap in Figure 4C showed the top 50 OTUs for total abundances associated with obesity and these OTU assignments were shown in Table S2. Most of OTUs, such as OUT684, OUT790, OUT777, OUT469, OUT810, OUT555, OUT625, OUT657, were positively correlated with body weight gain, blood lipids and negatively correlated with anti-inflammatory cytokines (Figure 4C). In particular, OTU479 and OUT545 were significantly positively correlated with the percent of epididymal fat weight, TG, TC, LDL and TNF-α, and negatively correlated with IL-10, and IL-4 levels, revealing that their changes were closely connected with changed obesity in rats.

There were notable differences between gut microbiota of HFD and ND groups from the taxonomic profiling at phylun and genus levels, and interventions with MN-Gup FM, MN-Gup + GOS FM and MN-Gup + XOS FM had influences on the gut microbiota composition induced by HFD (Figure S3). Most obviously, the relative abundance of *Bacteroidota* was significantly reduced by HFD, which was not reversed by interventions (Figure 4D). *Firmicutes*, the dominant phylum, were significantly elevated in the HFD group compared to the ND group, whereas its relative abundance was reduced by interventions, especially by MN-Gup + XOS FM with significance (*p* < 0.05, Figure 4E). At the genus level, the relative abundance of *Lachnoclostridium* and *Allobaculum* was found to be significantly increased in the HFD group, but significantly reduced by MN-Gup FM (*p* < 0.05, Figure 4F,G). These results indicate that fermented milk containing MN-Gup and its synbiotics with GOS and XOS could regulate HFD-affected gut microbiota and may alleviate obesity through the regulation of gut microbiota.

### 3.6. Effects of Fermented Milk Containing MN-Gup or MN-Gup-Based Synbiotics on the Profiles of SCFAs and BAs in HFD-Induced Obese Rats

**Figure 5:**
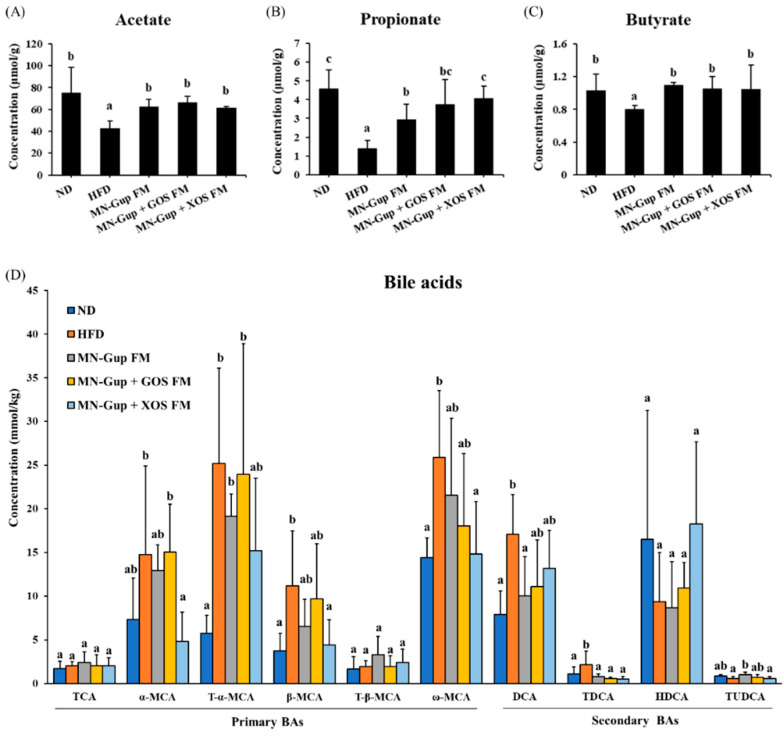
Effects of fermented milk containing MN-Gup or MN-Gup-based synbiotics on the profiles of short chain fatty acids (SCFAs) and bile acids (BAs) in HFD-induced obese rats. The concentration of (**A**) acetate, (**B**) propionate, and (**C**) butyrate was detected in colonic feces (*n* = 5). (**D**) The detectable bile acids in colonic feces, taurocholic acid (TCA), alpha-muricholic acid (α-MCA), tauro-alpha-muricholic acid (T-α-MCA), beta-muricholic acid (β-MCA), tauro-beta-muricholic acid (T-β-MCA), omega-muricholic acid (ω-MCA), deoxycholic acid (DCA), taurodeoxycholic acid (TDCA), hyodeoxycholic acid (HDCA) and tauroursodeoxycholic acid (TUDCA). Different lowercase letters (e.g., a, b, c) indicate significant differences, *p <* 0.05, (*n* = 4 in ND group, and *n* = 5 in the rest groups).

Gut microbiota-derived metabolites, such as SCFAs and BAs, have been considered as key molecular mediators between the microbiota and host. Gut microbiota can produce SCFAs via fermentation with dietary fiber, and modulate bile acid pool by metabolizing primary BAs to their secondary forms [6,27]. To give an insight into the potential mechanism associated with gut microbiota by which the interventions alleviated HFD-caused obesity, the levels of SCFAs and BAs were detected in colonic feces. As shown in Figure 5A–C, SCFAs, including acetate, propionate and butyrate, were significantly reduced by HFD feeding, which was significantly elevated by all interventions (*p* < 0.05), suggesting that fermented milk containing MN-Gup or MN-Gup-based synbiotics could promote the production of SCFAs.

Meanwhile, HFD feeding decreased the ratio of secondary BAs compared to ND feeding, revealing that HFD may suppress the biotransformation of primary BAs (Figure S4). MN-Gup FM and MN-Gup + GOS FM did not apparently change the ratio of secondary BAs, while an increased ratio of secondary BAs was observed in the MN-Gup + XOS FM group (Figure S4). Some primary BAs including α-MCA, T-α-MCA, β-MCA, and ω-MCA were significantly elevated in the HFD group, most of which were significantly reduced in the MN-Gup + XOS FM group (*p* < 0.05, Figure 5D). The levels of DCA and TDCA (secondary BAs) were significantly higher in the HFD group compared to the ND group (*p* < 0.05), but were lowered by all interventions. In contrast, HDCA and TUDCA (secondary BAs) levels were decreased in HFD, and interventions had inapparent reversions (Figure 5D). These results suggested that fermented milk containing MN-Gup or MN-Gup-based synbiotics could affect BAs levels and composition, and MN-Gup + XOS FM may show the best performance on regulating the HFD-changed bile acid pool.

## 4. Discussion

Given the high prevalence of obesity, finding an effective intervention to alleviate obesity is important for public health. Accumulating evidence demonstrates that the gut microbiota plays a crucial role in the development of obesity, and modifying gut microbiota through a diet rich in probiotics has become one of the most potential interventions for obesity [8]. Additionally, the synergism in the combination of prebiotics and probiotics has been attracting more and more attention due to the beneficial effects of prebiotic on gut microbiota and intestinal function [28]. *Bifidobacterium animalis* subsp. *lactis* MN-Gup (MN-Gup) has been found to regulate gut microbiota and relieve constipation [22], but its effects on obesity are still unknown. In this study, we evaluated the effects of MN-Gup-containing fermented milk on alleviating obesity using a therapeutic obesity model based on HFD-fed rats, and investigated whether prebiotics including GOS and XOS had the synergistic effects with MN-Gup on obesity.

The results showed that treatment with MN-Gup-containing fermented milk significantly reduced body weight gain and fat mass in HFD-induced obese rats (Figure 1). Although XOS and GOS could apparently promote the proliferation of MN-Gup in vitro (Figure S1), their supplement did not promote a more significant weight/fat-loss than MN-Gup alone in fermented milk, suggesting that they had no significant synergies with MN-Gup on reducing obesity (Figure 1). In fact, current opinions support that it is more important to make obese people healthier, as with improvements in blood lipids and chronic inflammation, rather than just focusing on body weight loss [29]. Similarly, MN-Gup FM, MN-Gup + GOS FM and MN-Gup + XOS FM could reduce the levels of TG, TC, LDL, TNF-α and IL-6, but GOS and XOS did not cause a significant synergistic effect with MN-Gup (Figure 2 and Figure 3). Pro-inflammatory cytokines, such as TNF-α and IL-6, could contribute to systemic insulin resistance or metabolic disorder in obesity [30], while anti-inflammatory cytokines, such as IL-10 and IL-4, could prevent diet-induced obesity and insulin resistance [31,32]. In particular, MN-Gup + XOS FM did not significantly reverse the blood lipids and pro-inflammatory cytokines, which was not as effective as the MN-Gup alone and MN-Gup + GOS FM. However, MN-Gup + XOS FM significantly enhanced the level of IL-10 and IL-4 (Figure 3), revealing that XOS may have had a promotion on health perhaps in a unique way. These results suggested that prebiotics did not always have synergistic effects with probiotics, and it is necessary to confirm the effectiveness of prebiotics for probiotic strains through more experimental data including animal and clinical data before commercial use.

In previous studies, gut microbiota showed differences in obese individuals compared to normal individuals [33]. The results from PCoA (Figure 4A) and taxonomic composition (Figure S3) analysis showed that HFD feeding apparently altered the composition of gut microbiota in rats, which was consistent with previous reports [34,35]. Numerous OTUs were found to correlate with the obesity-related indexes (Figure 4C). Notably, OTU479 and OTU545, which were respectively annotated as *Enterococcus* and *Lachnoclostridium* genus (Table S2), were significantly positively correlated with most phenotypes pro-obesity and were negatively correlated with the anti-obesity phenotypes (Figure 4C). *Enterococcus* and *Lachnoclostridium* have been previously reported to be elevated in HFD-fed mice and their increases were associated with obesity and related inflammation [36,37]. At the phylum level, the *Bacteroidota* proportion was largely reduced by HFD and was hardly changed by interventions (Figure 4D), suggesting that a long-term high fat diet could cause an irreversible effect on *Bacteroidota*. A significant increase of *Firmicutes* proportion was observed in the HFD group, and only MN-Gup FM could significantly reverse it (Figure 4E), revealing that a GOS and XOS supplement may benefit the growth of *Firmicutes*. At the genus level, the relative abundance of *Lachnoclostridium* was significantly elevated by HFD and reversed by the interventions (Figure 4F), which was consistent with the correlation analysis (Figure 4C). Additionally, similar results were also observed in the *Allobaculum* proportion (Figure 4G). *Allobaculum* has been demonstrated to be increased in HFD and involved in lipid metabolism and fat deposition [38]. These results indicated that fermented milk containing MN-Gup and MN-Gup based synbiotics may attenuate HFD-induced obesity through the regulation of gut microbiota.

Previous research has considered that one potential mechanism underlying the regulation of obesity by gut microbiota is through microbiome-derived bioactive metabolites like SCFAs and BAs and their regulatory effects on the host metabolism [39,40]. The major SCFAs, including acetate, propionate, and butyrate, have been demonstrated to reduce lipogenesis and inflammation [6], and their significant increases in MN-Gup FM, MN-Gup + GOS FM and MN-Gup + XOS FM (Figure 5A–C) may be the explanations for the alleviation of obesity. Interestingly, MN-Gup + XOS FM led to significantly higher propionate concentration than MN-Gup FM and MN-Gup + GOS FM (Figure 5B). Propionate was found to promote the secretion of a gut hormone related to energy metabolism and to regulate appetite via the gut-brain axis [6]. It was speculated that XOS may benefit the growth of propionate-producing bacteria, which may help to alleviate obesity.

Recent studies had also shown that gut microbiota could metabolize primary BAs to secondary Bas, and that BAs levels and composition are associated with metabolic diseases such as obesity, dyslipidemia, and diabetes [41]. Our results indicated that HFD decreased the proportion of secondary BAs compared to ND (Figure S4), which may result from the HFD-altered gut microbiota. Notably, significant increases of muricholic acids (including α-MCA, T-α-MCA, β-MCA, and ω-MCA) were observed in the HFD group, which were consistent with previous studies [42], and revealed the suppression of primary BAs metabolism. MN-Gup + XOS FM showed significantly lower concentrations of α-MCA, T-α-MCA, β-MCA, and ω-MCA than the HFD group (Figure 5D), suggesting its potential role in regulation lipid and glucose metabolism. Moreover, DCA and its conjugated form TDCA have been demonstrated to trigger the production of proinflammatory cytokines like TNF-α and IL-6 [42,43], so their significant increase in HFD and decrease in the intervention groups (especially MN-Gup + XOS FM) were consistent with the obesity-related inflammation and the alleviation by interventions. Taken together, fermented milk containing MN-Gup or MN-Gup-based synbiotics may alleviate HFD-induced obesity through microbiome-associated SCFAs and BAs. In the MN-Gup-based synbiotics, MN-Gup + GOS FM and MN-Gup + XOS FM had their own advantages and characteristics in the regulation of gut microbiota and SCFAs and BAs.

## 5. Conclusions

In conclusion, our study demonstrated that fermented milk containing MN-Gup or its synbiotics (MN-Gup + GOS, MN-Gup + XOS) had the potential effects on alleviating obesity in HFD-induced obese rats, as shown by the decreases in body weight gain, epididymal fat mass, adipocyte sizes and the improvement in dyslipidemia and obesity-related inflammation. Furthermore, they could regulate the HFD-affected gut microbiota through lowing obesity-related bacteria like *Firmicutes*, *Lachnoclostridium* and *Allobaculum*, and modify the microbiota-driven metabolites including SCFAs and BAs, which may be the potential mechanism for obesity alleviation. Although GOS and XOS could promote the growth of MN-Gup in vitro, in vivo results did not show that GOS and XOS had significant synergistic effects with MN-Gup on alleviating obesity, revealing that the symbiotic applications based on MN-Gup will need more research and evidence. This study will provide the underpinning for the future clinical trial of MN-Gup and scientific supports for applications of MN-Gup in probiotic products.

## Data Availability

Data are contained within the article or Supplementary Materials.

## Supplementary Materials

The following supporting information can be downloaded at: https://www.mdpi.com/article/10.3390/nu14132631/s1, Figure S1: Screening prebiotics with the capability to promote the growth of MN-Gup in vitro. (A)The growth curve of MN-Gup in 24 h; and (B) Prebiotic index (PI) of prebiotics. FOS, fructo-oligosaccharides; RD, resistance dextrin; XOS, xylo-oligosaccharides; GOS, galacto-oligosaccharides; Figure S2: (A) The body weight of rats before high-fat diet (HFD) feeding, and the beginning of end of interventions; (B) The intake of feed calories during intervention period (*n* = 5). ND, normal diet; HFD, fed high-fat diet; MN-Gup FM, fermented milk containing MN-Gup; MN-Gup FM + GOS, fermented milk containing MN-Gup and galacto-oligosaccharides (GOS); MN-Gup FM + XOS, fermented milk containing MN-Gup and xylo-oligosaccharides (XOS); Figure S3: The average relative abundance of bacteria at (A) phylum and (B) genus level (*n* = 5). ND, normal diet; HFD, fed high-fat diet; MN-Gup FM, fermented milk containing MN-Gup; MN-Gup FM + GOS, fermented milk containing MN-Gup and galacto-oligosaccharides (GOS); MN-Gup FM + XOS, fermented milk containing MN-Gup and xylo-oligosaccharides (XOS); Figure S4: Effects of fermented milk containing MN-Gup or MN-Gup-based synbiotics on the proportion of primary and secondary bile acids (BAs) in colonic feces. ND, normal diet; HFD, fed high-fat diet; MN-Gup FM, fermented milk containing MN-Gup; MN-Gup FM + GOS, fermented milk containing MN-Gup and galacto-oligosaccharides (GOS); MN-Gup FM + XOS, fermented milk containing MN-Gup and xylo-oligosaccharides (XOS) (*n* = 4 in ND group, and *n* = 5 in the rest groups); Table S1: The tested bile acids and their abbreviation; Table S2: OTU assignments in the correlation analysis.
