# Supplementary material for: Effects of Fermented Milk Containing Bifidobacterium animalis Subsp. lactis MN-Gup (MN-Gup) and MN-Gup-Based Synbiotics on Obesity Induced by High Fat Diet in Rats
Source: Nutrients. 2022 Jun 24;14(13):2631. doi: 10.3390/nu14132631 (PMC9268376; doi:10.3390/nu14132631)
Supplement: Supplementary file 1 [file nutrients-14-02631-s001.zip › Supplementary Materials-the final version.pdf]

Supplementary Materials

Effects of fermented milk containing *Bifidobacterium animalis* subsp. *lactis* MN-Gup (MN-Gup) and MN-Gup-based synbiotics on obesity induced by high fat diet in rats

Chenyuan Wang[1,2], Shusen Li[2], Erna Sun[2], Ran Xiao[1,2], Ran Wang[3], Yimei Ren[1], Jingjing He[1], Qi Zhang[1], Jing Zhan[1,*]

[1] Key Laboratory of Precision Nutrition and Food Quality, Department of Nutrition and Health, China Agricultural University, Beijing 100193, China P.R.

[2] Mengniu Hi-tech Dairy Product Beijing Co., Ltd., Beijing 101100 China P.R.

[3] Key Laboratory of Functional Dairy, Department of Nutrition and Health, China Agricultural University, Beijing 100193, China P.R.

***** Correspondence:

Jing Zhan, Key Laboratory of Precision Nutrition and Food Quality, Department of Nutrition and Health, China Agricultural University, No.2 West Yuanmingyuan Road, Beijing 100193, China P.R.

Tel/Fax: +86 010 62738589;

Email address: jingzhan@cau.edu.cn

Table S1 The tested bile acids and their abbreviation

Bile acids	Abbreviation
Cholic acid	CA
Lithocholic acid	LCA
Ursodeoxycholic acid	UDCA
Chenodeoxycholic acid	CDCA
Deoxycholic acid	DCA
Hyodeoxycholic acid	HDCA
Alpha-Muricholic acid	α-MCA
Beta-Muricholic acid	β-MCA
Gamma-Muricholic acid	γ-MCA
Omega-Murichoclic acid	ω-MCA
Glycocholic acid	GCA
Glycodeoxycholic acid	GDCA
Glycolithocholic acid	GLCA
Glycochenodeoxycholic acid	GCDCA
Glycohyodeoxycholic acid	GHDCA
Glycoursodeoxycholic acid	GUDCA
Taurocholic acid	TCA
Taurolithocholic acid	TLCA
Taurochenodeoxycholic acid	TCDCA
Tauro-alpha-Muricholic acid	T-α-MCA
Tauro-beta-Muricholic acid	T-β-MCA
Taurodeoxycholate acid	TDCA
Tauroursodeoxycholic acid	TUDCA
Taurohyodeoxycholic acid	THDCA

(A)

(B)

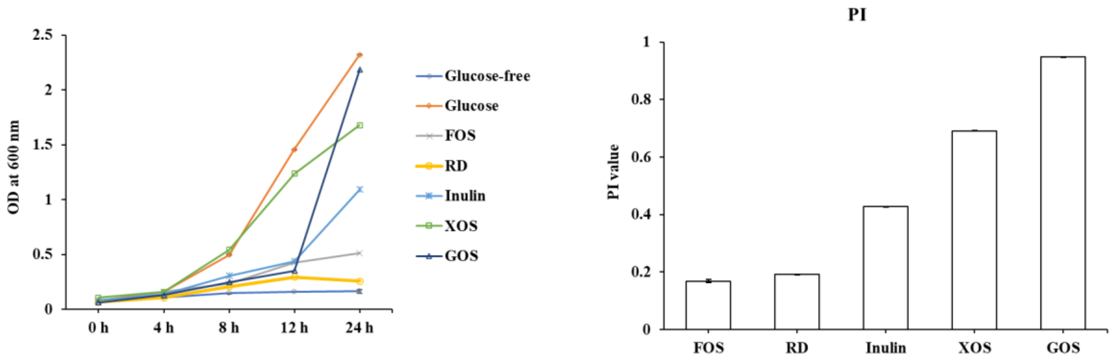

Figure S1. Screening prebiotics with the capability to promote the growth of MN-Gup *in vitro* (n = 3). (A)The growth curve of MN-Gup in 24 hours; and (B) Prebiotic index (PI) of prebiotics. FOS, fructo-oligosaccharides; RD, resistance dextrin; XOS, xylo-oligosaccharides; GOS, galacto-oligosaccharides.

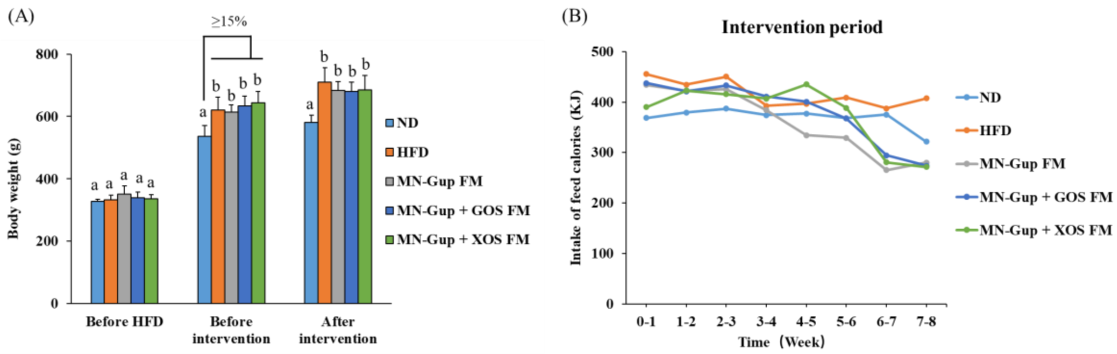

Figure S2. (A) The body weight of rats before high-fat diet (HFD) feeding, and the beginning of end of interventions; (B) The intake of feed calories duringintervention period (n = 5). Different lowercase letters indicate significant differences, $p < 0.05$. ND, normal diet; HFD, fed high-fat diet; MN-Gup FM, fermented milk containing MN-Gup; MN-Gup FM+GOS, fermented milk containing MN-Gup and galacto-oligosaccharides (GOS); MN-Gup FM+XOS, fermented milk containing MN-Gup and xylo-oligosaccharides (XOS).

Table S2 OTU assignments in the correlation analysis

OTUs	Phylum	Class	Order	Family	Genus	Species
OTU784	Desulfobacterota	Desulfovibrionia	Desulfovibrionales	Desulfovibrionaceae	Desulfovibrio	Desulfovibrio_fairfieldensis
OTU748	Firmicutes	Clostridia	Lachnospirales	Lachnospiraceae	Eubacterium_xylanophilum_group	uncultured_bacterium_g_Eubacterium_xylanophilum_group
OTU527	Firmicutes	Clostridia	Clostridia_UCG-014	norank_o_Clostridia_UCG-014	norank_f_norank_o_Clostridia_UCG-014	uncultured_organism_g_norank_f_norank_o_Clostridia_UCG-014
OTU675	Firmicutes	Clostridia	Oscillospirales	Eubacterium_coprostanoligenes_group	norank_f_Eubacterium_coprostanoligenes_group	uncultured_bacterium_g_norank_f_Eubacterium_coprostanoligenes_group
OTU709	Firmicutes	Clostridia	Lachnospirales	Lachnospiraceae	Blautia	
OTU618	Firmicutes	Clostridia	Lachnospirales	Lachnospiraceae	Blautia	
OTU134	Patescibacteria	Saccharimonadia	Saccharimonadales	Saccharimonadaceae	Candidatus_Saccharimonas	uncultured_bacterium_g_Candidatus_Saccharimonas
OTU465	Firmicutes	Clostridia	Peptostreptococcales-Tissierellales	Anaerovoracaceae	Eubacterium_nodatum_group	
OTU102	Actinobacteriota	Coriobacteriia	Coriobacteriales	Eggerthellaceae	Adlercreutzia	uncultured_bacterium_g_Adlercreutzia
OTU426	Firmicutes	Clostridia	Lachnospirales	Lachnospiraceae	Blautia	
OTU573	Firmicutes	Clostridia	Oscillospirales	Oscillospiraceae	NK4A214_group	uncultured_rumen_bacterium_g_NK4A214_group
OTU344	Actinobacteriota	Coriobacteriia	Coriobacteriales	Eggerthellaceae	norank_f_Eggerthellaceae	uncultured_bacterium_g_norank_f_Eggerthellaceae
OTU684	Firmicutes	Clostridia	Lachnospirales	Lachnospiraceae	Sellimonas	uncultured_organism_g_Sellimonas
OTU548	Fusobacteriota	Fusobacteriia	Fusobacteriales	Fusobacteriaceae	Fusobacterium	Fusobacterium_russii
OTU22	Firmicutes	Bacilli	Lactobacillales	Lactobacillaceae	Lactobacillus	Lactobacillus_johnsonii
OTU788	Firmicutes	Clostridia	Lachnospirales	Lachnospiraceae	norank_f_Lachnospiraceae	butyrate-producing_bacterium_L2-10
OTU769	Firmicutes	Bacilli	Lactobacillales	Streptococcaceae	Streptococcus	Streptococcus_respiraculi
OTU629	Firmicutes	Clostridia	Christensenellales	Christensenellaceae	Christensenellaceae_R-7_group	uncultured_prokaryote_g_Christensenellaceae_R-7_group

OTU719	Firmicutes	Clostridia	Lachnospirales	Lachnospiraceae	norank_f__Lachnospiraceae	unclassified_g_norank_f_Lachnospiraceae
OTU717	Firmicutes	Clostridia	Lachnospirales	Lachnospiraceae	Dorea	uncultured_bacterium_g_Dorea
OTU11	Firmicutes	Bacilli	Erysipelotrichales	Erysipelotrichaceae	Turicibacter	uncultured_bacterium_g_Turicibacter
OTU5	Actinobacteriota	Actinobacteria	Bifidobacteriales	Bifidobacteriaceae	Bifidobacterium	Bifidobacterium_animalis
OTU553	Firmicutes	Bacilli	Lactobacillales	Streptococcaceae	Streptococcus	Streptococcus_hyointestinalis
OTU763	Actinobacteriota	Actinobacteria	Micrococcales	Micrococcaceae	Rothia	
OTU595	Firmicutes	Bacilli	Lactobacillales	Lactobacillaceae	Lactobacillus	Lactobacillus_reuteri
OTU790	Firmicutes	Clostridia	Lachnospirales	Lachnospiraceae	norank_f_Lachnospiraceae	
OTU453	Firmicutes	Bacilli	Staphylococcales	Staphylococcaceae	Staphylococcus	Staphylococcus_nepalensis
OTU777	Firmicutes	Clostridia	Lachnospirales	Lachnospiraceae	Blautia	
OTU814	Firmicutes	Clostridia	Lachnospirales	Lachnospiraceae	Ruminococcus_torques_group	uncultured_bacterium_g_Ruminococcus_torques_group
OTU469	Firmicutes	Clostridia	Lachnospirales	Lachnospiraceae	Lachnospiraceae_UCG-010	uncultured_bacterium_g_Lachnospiraceae_UCG-010
OTU570	Firmicutes	Clostridia	Oscillospirales	Eubacterium_coprostanoligenes_group	norank_f_Eubacterium_coprostanoligenes_group	gut_metagenome_g_norank_f_Eubacterium_coprostanoligenes_group
OTU741	Firmicutes	Clostridia	Lachnospirales	Lachnospiraceae	Ruminococcus_gauvreauii_group	uncultured_organism_g_Ruminococcus_gauvreauii_group
OTU810	Firmicutes	Clostridia	Lachnospirales	Lachnospiraceae		
OTU479	Firmicutes	Bacilli	Lactobacillales	Enterococcaceae	Enterococcus	Enterococcus_faecium_g_Enterococcus
OTU620	Firmicutes	Clostridia	Lachnospirales	Lachnospiraceae	Lachnospiraceae_NK4A136_group	
OTU545	Firmicutes	Clostridia	Lachnospirales	Lachnospiraceae	Lachnoclostridium	
OTU555	Firmicutes	Clostridia	Lachnospirales	Lachnospiraceae	Blautia	
OTU79	Firmicutes	Bacilli	Lactobacillales	Lactobacillaceae	Lactobacillus	Lactobacillus_murinus
OTU696	Firmicutes	Bacilli	Lactobacillales	Streptococcaceae	Streptococcus	

OTU621	Firmicutes	Clostridia	Lachnospirales	Lachnospiraceae	Blautia	uncultured_Lachnospiraceae_bacterium_g_Blautia
OTU644	Actinobacteriota	Coriobacteriia	Coriobacteriales	Coriobacteriaceae	Collinsella	Collinsella_provencensis
OTU452	Firmicutes	Clostridia	Clostridiales	Clostridiaceae	Clostridium_sensu_stricto_1	
OTU577	Firmicutes	Clostridia	Lachnospirales	Lachnospiraceae	Lachnospiraceae_NK4A136_group	uncultured_bacterium_g_Lachnospiraceae_NK4A136_group
OTU635	Firmicutes	Bacilli	Erysipelotrichales	Erysipelotrichaceae	Allobaculum	
OTU450	Firmicutes	Clostridia	Lachnospirales	Lachnospiraceae		
OTU14	Firmicutes	Clostridia	Oscillospirales	Oscillospiraceae	UCG-005	uncultured_bacterium_g_UCG-005
OTU625	Firmicutes	Bacilli	Erysipelotrichales	Erysipelotrichaceae	Allobaculum	
OTU653	Proteobacteria	Gammaproteobacteria	Enterobacterales	Enterobacteriaceae	Escherichia-Shigella	Escherichia_coli_g_Escherichia-Shigella
OTU657	Firmicutes	Clostridia	Lachnospirales	Lachnospiraceae	Blautia	
OTU427	Firmicutes	Clostridia	Peptostreptococcales-Tissierellales	Peptostreptococcaceae	Romboutsia	Romboutsia_ilealis

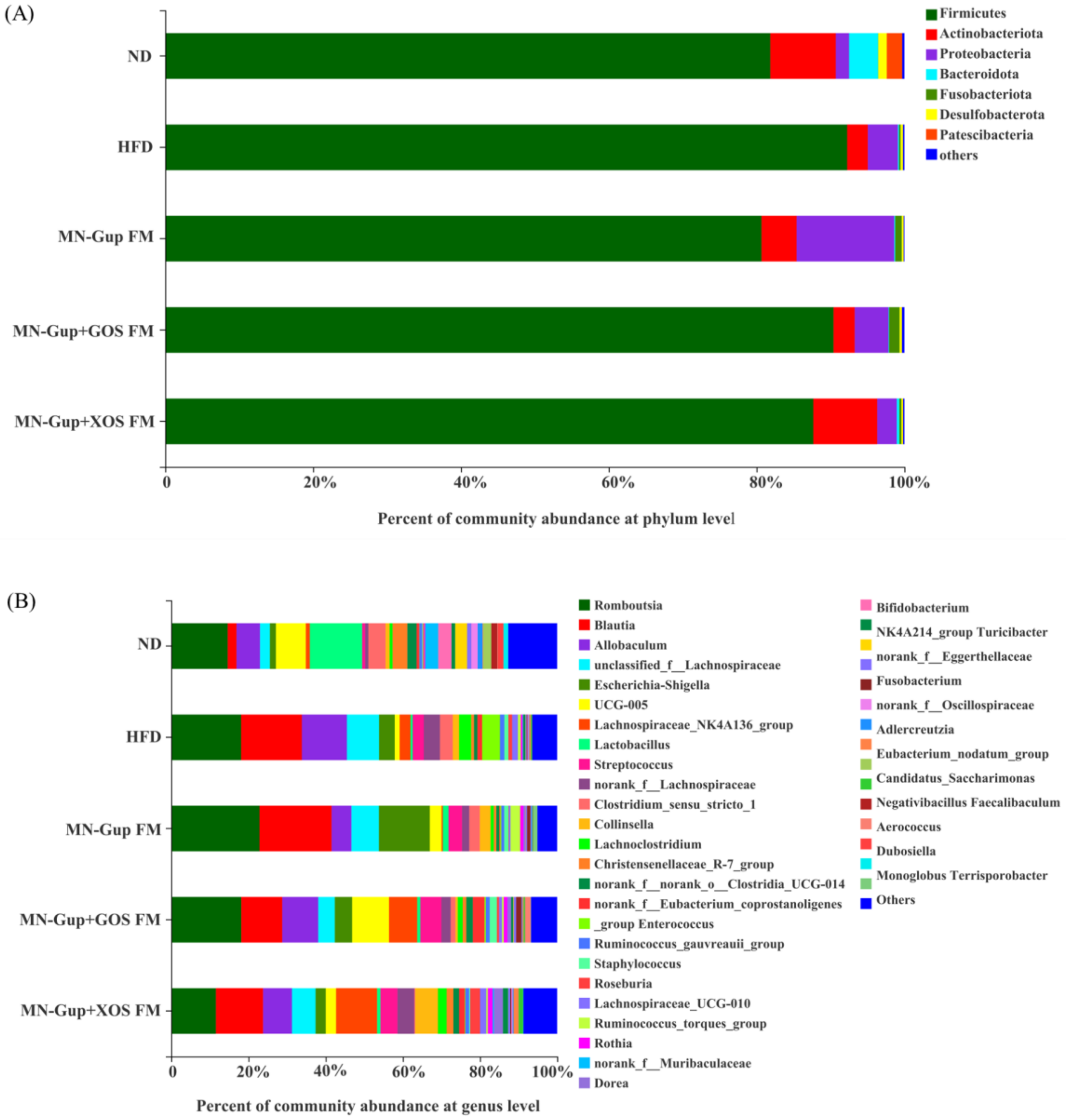

Figure S3. The average relative abundance of bacteria at (A) phylum and (B) genus level (*n* = 5). ND, normal diet; HFD, fed high-fat diet; MN-Gup FM, fermented milk containing MN-Gup; MN-Gup FM+GOS, fermented milk containing MN-Gup and galacto-oligosaccharides (GOS); MN-Gup FM+XOS, fermented milk containing MN-Gup and xylo-oligosaccharides (XOS).

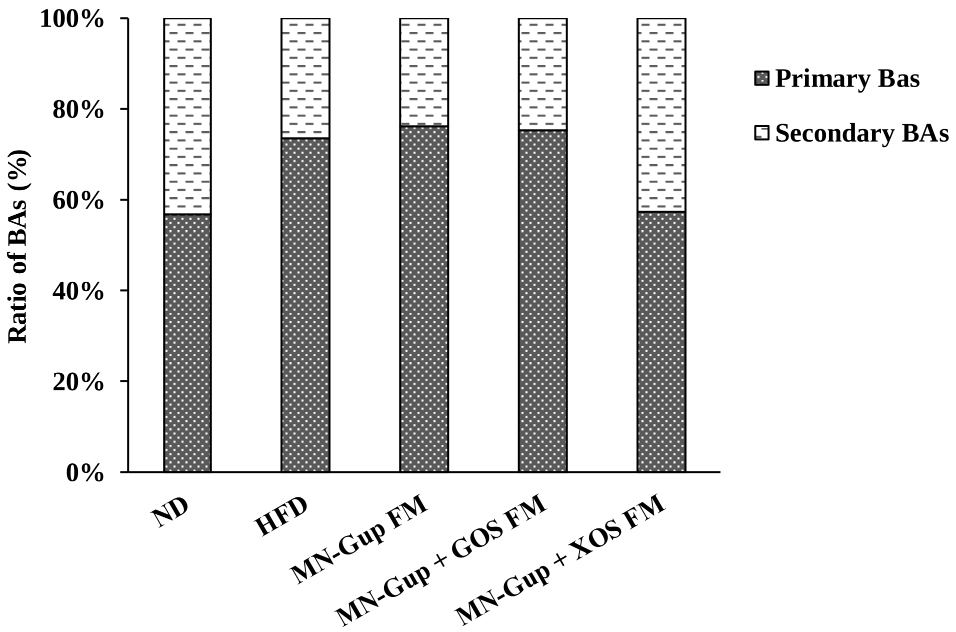

Figure S4. Effects of fermented milk containing MN-Gup or MN-Gup-based synbiotics on the proportion of primary and secondary bile acids (BAs) in colonic feces. ND, normal diet; HFD, fed high-fat diet; MN-Gup FM, fermented milk containing MN-Gup; MN-Gup FM+GOS, fermented milk containing MN-Gup and galacto-oligosaccharides (GOS); MN-Gup FM+XOS, fermented milk containing MN-Gup and xylo-oligosaccharides (XOS) (n = 4 in ND group, and n = 5 in the rest groups).
